# The Association Between Remnant Cholesterol and the Estimated 10-Year Risk of a First Hard Cardiovascular Event

**DOI:** 10.3389/fcvm.2022.913977

**Published:** 2022-06-17

**Authors:** Zhen Yang, Kuo Yang, Junhe Shi, Qiaoning Yang, Ying Zhang, Jie Gao, Dazhuo Shi, Hua Qu

**Affiliations:** ^1^Xiyuan Hospital, China Academy of Chinese Medical Sciences, Beijing, China; ^2^Cardiovascular Department, Peking University Traditional Chinese Medicine Clinical Medical School (Xiyuan), Beijing, China; ^3^Academy of Integration of Chinese and Western Medicine, Peking University Health Science Center, Beijing, China; ^4^National Clinical Research Center for Chinese Medicine Cardiology, Beijing, China; ^5^School of Computer and Information Technology, Beijing Jiaotong University, Beijing, China; ^6^National Medical Products Administration, Key Laboratory for Clinical Research and Evaluation of Traditional Chinese Medicine, Beijing, China

**Keywords:** Remnant cholesterol, 10-year risk, atherosclerotic cardiovascular disease, NHANES, cross-sectional study

## Abstract

**Background:**

Remnant cholesterol (Remnant-C), rather than TG, is believed to increase the risk of atherosclerotic cardiovascular disease. We evaluated whether Remnant-C is associated with an estimated 10-year risk of a first hard atherosclerotic cardiovascular disease event.

**Methods:**

This cross-sectional study was performed on 2,048 participants (1,130 men and 918 women), aged 40 to 79 years, from the National Health and Nutrition Examination Survey (NHANES) between 1999 and 2018. The independent variable was Remnant-C; the dependent variable was the 10-year risk of a first hard atherosclerotic cardiovascular disease event (defined as non-fatal myocardial infarction, coronary heart disease death, or stroke, over a 10-year period among people free from atherosclerotic cardiovascular disease at the beginning of the period). The other variables, such as smoking behavior, hypertension, diabetes etc., were considered as the potential effect modifiers. Multivariate linear regression models and smooth curve fittings were used to evaluate the association between Remnant-C and the 10-year risk of a first hard atherosclerotic cardiovascular disease event. Subgroup analyses stratified by gender and race were also performed.

**Results:**

A positive association between Remnant-C and the 10-year risk of a first hard atherosclerotic cardiovascular disease event was demonstrated in the fully adjusted model (β = 0.078, [95%CI: 0.061–0.094], *P* < 0.001). The 10-year risk was increased by 0.078% for each 1 mg/dl increase in Remnant-C. In the subgroup analysis for gender, this association remained in both men (β = 0.128, [95%CI: 0.108–0.148], *P* < 0.001) and women (β = 0.043, [95%CI: 0.016–0.070], *P* = 0.00179). However, in the subgroup analysis for race, the association between Remnant-C and the 10-year risk reached an inflection point at remnant-C 38 mg/dL, where a positive association was not as obvious for the non-Hispanic Black population as for other racial groups.

**Conclusions:**

Remnant-C positively correlates with a 10-year risk of a first hard atherosclerotic cardiovascular disease event.

## Introduction

Dyslipidemia is an important causal factor for atherosclerotic cardiovascular disease (ASCVD). Among them, low-density lipoprotein cholesterol (LDL-C) has been regarded as a factor most closely associated with the occurrence and development of ASCVD. The risk of ASCVD remains high after treatment with LDL-C lowering drugs, including statins and other lipid-lowering drugs to decrease LDL-C to a relatively low level (1). Currently, mixed dyslipidemia, which commonly occurs in the patients with type II diabetes mellitus and metabolic syndrome, has raised serious concern (2). Mixed dyslipidemia is characterized by hypertriglyceridemia, low high-density lipoprotein cholesterol (HDL-C), a preponderance of small, dense LDL particles, and cholesterol-rich remnant particles, which has newly emerged as the biggest “competitor” for LDL-C among lipid risk factors for cardiovascular disease (3). An elevated plasma triglyceride (TG) level presents in increased chylomicron (CM) or very low-density lipoprotein (VLDL) particles. CM and VLDL are regarded as triglyceride-rich lipoproteins (TRL), and the metabolized TRL is named remnant lipoprotein particle (RLP) with a high triglyceride concentration. Emerging evidence has shown that the cholesterol component of RLP, the remnant cholesterol (Remnant-C), rather than TG, increases the risk of ASCVD (4).

Remnant-C, including the cholesterol of VLDL and intermediate-density lipoprotein residues in a fasting state, and the cholesterol of CM residues in a post-prandial state, is the total non-LDL and non-HDL cholesterol (5). Studies have shown that Remnant-C has a predictive value for the risk of ASCVD. A study of metabonomics analyses showed that statins significantly reduced Remnant-C levels (equivalent to an 80% reduction in LDL-C), but only slightly reduced TG (equivalent to a 25% reduction in LDL-C). Pravastatin reduced Remnant-C more obviously than LDL-C (6, 7). In a recent prospective study involving 2,973 patients who had suffered acute myocardial infarctions or ischemic strokes, patients with Remnant-C <0.8 mmol/L were found to have a 20% reduction in recurrent cardiovascular events, suggesting that elevated levels of Remnant-C increase the risk of cardiovascular events and are a better predictor than LDL-C (8). However, LDL-C measurement is still applied in clinical practice for people with daily physical examination screening for cardiovascular diseases, and Remnant-C has not been used as a prospective predictor yet. Also, Remnant-C prediction of cardiovascular events in patients without coronary heart disease has not been reported to date.

Here, we conducted a cross-sectional study to estimate the specific value of the remnant cholesterol for the 10-year risk of a first hard ASCVD event (defined as non-fatal myocardial infarction, coronary heart disease death, or fatal or non-fatal stroke, over a 10-year period among people free from ASCVD at the beginning of the period) according to the 2013 ACC/AHA guideline on the Assessment of Cardiovascular Risk using a large-scale database from National Health and Nutrition Examination Survey (NHANES) (9).

## Methods

### Study Population

The NHANES is a population-based national survey that collected information on the health and nutrition in the United States in biennial cycles. The survey data are publicly available on the internet for data users and researchers worldwide. Full details of the design and operation are available at www.cdc.gov/nchs/nhanes/. Our analysis was based on data from 1999–2018, which represent 10 cycles of the NHANES. We evaluated the risk of a first hard ASCVD event in adults aged from 40 to 79 years without existing ASCVD disease according to the 2013 ACC/AHA guidelines. Eligible participants were 40 to 79 years of age; had no ASCVD disease history; had HDL-C of 20–100 mg/dl; and had total cholesterol (TC) of 130–320 mg/dl and systolic blood pressure (SBP) of 90–200 mmHg. In the final analysis, 2,048 participants were enrolled after those who were excluded for missing Remnant-C data (*n* = 2334). The ethics review board of the National Center for Health Statistics approved all NHANES protocols and written informed consent was obtained from all participants (10).

### Variables

Continuous variables included age, body mass index (BMI), SBP, diastolic blood pressure (DBP), TC, TG, HDL-C and LDL-C. Categorical variables included gender, race/ethnicity, smoking behavior, hypertension treatment (treated or not), and diabetes. The remnant cholesterol was calculated according to the equation: Remnant-C = TC — HDL-C — LDL-C (11). The 10-year risk of a first hard ASCVD event was calculated according to the 2013 ACC/AHA Guideline on the Assessment of Cardiovascular Risk, which can be accessed by https://tools.acc.org/ASCVD-Risk-Estimator-Plus/#!/calculate/estimate/.

### Statistical Analysis

Continuous variables were expressed as mean and standard deviation (SD) values if the data were normally distributed, or as median values and interquartile ranges otherwise; comparisons between groups were analyzed with the *t*-test if the data were normally distributed, or using the Mann-Whitney test if the data were not normally distributed. Categorical variables were described as percentages and compared by χ2 testing. Multivariable logistic regression was used to assess the association between Remnant-C and the 10-year risk. Subgroup analyses were performed to test the stratified associations between Remnant-C and the 10-year risk of a first hard ASCVD. All statistical analyses were performed with R (The R Foundation; http://www.R-project.org; version 3.4.3) and EmpowerStats software (http://www.empowerstats.com, X&Y Solutions, Inc., Boston, MA).

## Results

### Study Participant Characteristics

In our analysis, 2,048 participants were enrolled, with the characteristics of the participants subclassified based on Remnant-C quartiles (Q1: 5.0–17.0 mg/dL; Q2: 18.0–23.0 mg/dL; Q3: 24.0–34.0 mg/dL; and Q4: 35.0–79.0 mg/dL), as shown in Table 1. There were significant differences in baseline data between the Remnant-C quartiles, with the exception of gender, smoker status, SBP, and hypertension treatment. Men were present in higher percentages than women in all four Remnant-C quartile groups (Q1, 54.49%; Q2, 52.75%; Q3, 54.93%; Q4, 58.18%), especially in the highest Remnant-C quartile subgroup. The 10-year risk of a first hard ASCVD event increased along with the Remnant-C increase (*P* = 0.002).

**Table 1:** Baseline characteristic of participants.

**Remnant-C (mg/dL)**	**Q1 (5.0–17.0)**	**Q2 (18.0–23.0)**	**Q3 (24.0–34.0)**	**Q4 (35.0–79.0)**	***P*-value**
**Gender**					0.364
Men	267 (54.49%)	249 (52.75%)	301 (54.93%)	313 (58.18%)	
Women	223 (45.51%)	223 (47.25%)	247 (45.07%)	225 (41.82%)	
Age (years)	61.38 ± 10.18	60.59 ± 9.90	60.18 ± 10.21	59.36 ± 10.31	0.014
**Race**					<0.001
Non-Hispanic Black	201 (41.02%)	136 (28.81%)	106 (19.34%)	62 (11.52%)	
Other	289 (58.98%)	336 (71.19%)	442 (80.66%)	476 (88.48%)	
BMI (kg/m^2^)	29.58 ± 7.36	30.02 ± 6.56	31.40 ± 6.57	31.31 ± 5.82	<0.001
**Smoking**					0.197
Yes	154 (31.43%)	169 (35.81%)	195 (35.58%)	203 (37.73%)	
No	336 (68.57%)	303 (64.19%)	353 (64.42%)	335 (62.27%)	
SBP (mmHg)	133.01 ± 19.09	132.55 ± 18.56	132.70 ± 19.71	134.45 ± 19.22	0.356
DBP (mmHg)	70.77 ± 14.64	72.21 ± 13.55	72.15 ± 13.47	74.51 ± 13.77	<0.001
**Hypertension treatment**					0.751
Yes	430 (87.76%)	406 (86.02%)	470 (85.77%)	461 (85.69%)	
No	60 (12.24%)	66 (13.98%)	78 (14.23%)	77 (14.31%)	
**Diabetes**					<0.001
Yes	99 (20.20%)	101 (21.40%)	159 (29.01%)	160 (29.74%)	
No	391 (79.80%)	371 (78.60%)	389 (70.99%)	378 (70.26%)	
TC (mg/dL)	186.34 ± 32.32	195.14 ± 34.65	199.13 ± 35.01	209.91 ± 37.85	<0.001
HDL-C (mg/dL)	61.96 ± 14.72	56.54 ± 13.81	49.60 ± 12.13	43.91 ± 11.40	<0.001
TG (mg/dL)	67.72 ± 13.40	102.03 ± 8.79	142.17 ± 15.25	239.89 ± 54.41	<0.001
LDL-C (mg/dL)	110.85 ± 30.17	118.17 ± 31.41	121.10 ± 32.87	118.02 ± 35.59	<0.001
10-year risk (%)	15.81 ± 12.67	15.31 ± 12.26	17.04 ± 13.82	17.96 ± 13.29	0.002

*Mean ± SD for continuous variables. (%) for categorical variables. Remnant-C, remnant cholesterol. BMI, body mass index; SBP, systolic blood pressure; DBP, diastolic blood pressure; TC, total cholesterol; HDL-C, high-density lipoprotein cholesterol; TG, triglyceride; LDL-C, low density lipoprotein cholesterol*.

### Associations Between Remnant-C and the 10-Year Risk of a First Hard ASCVD Event

**Figure 1:**
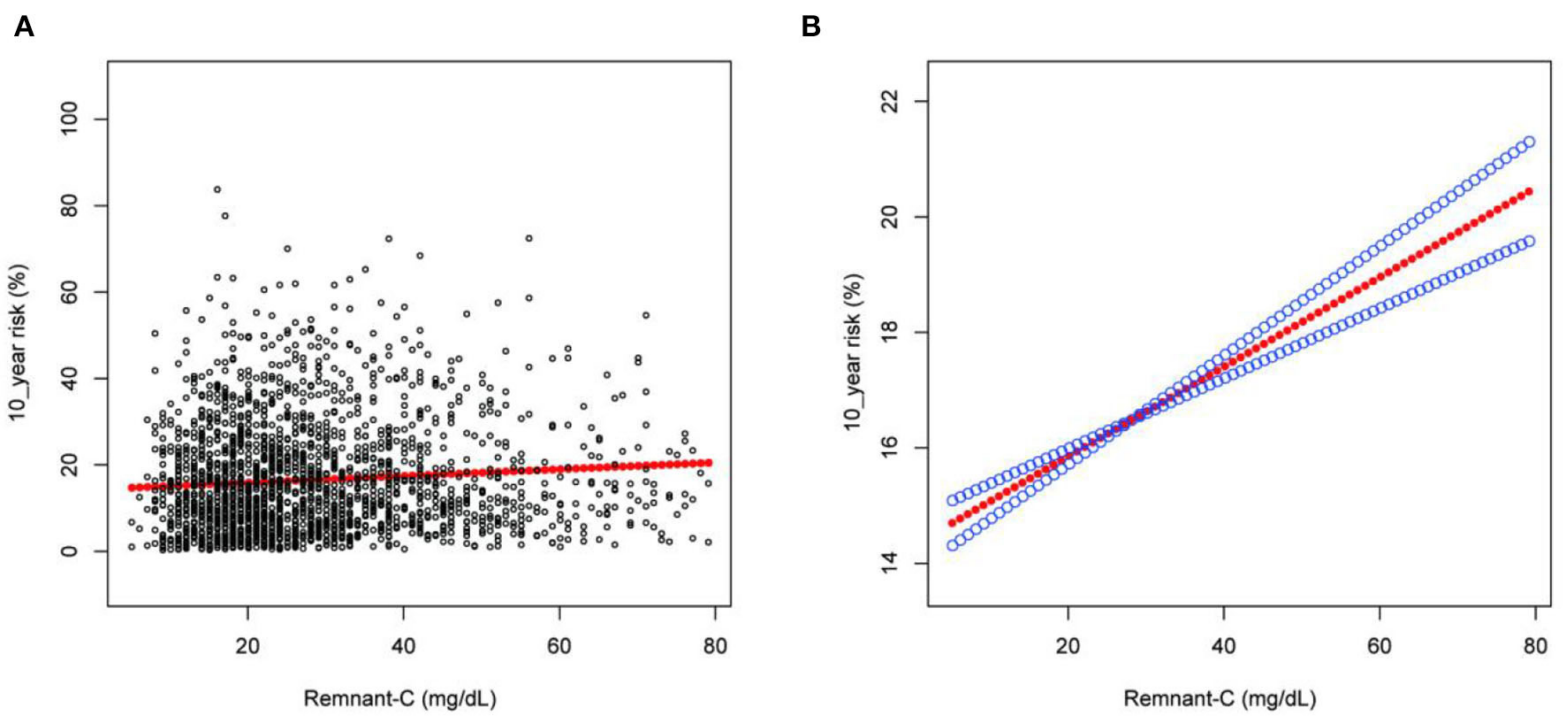
The association between Remnant-C and the 10-year risk of a first hard ASCVD event. **(A)** Each black point represents a sample. **(B)** Solid red line represents the smooth curve fit between variables. Blue lines represent the 95% of confidence interval from the fit. Gender, age, race, body mass index, systolic blood pressure, diastolic blood pressure, hypertension treatment, smoking, diabetes, and low-density lipoprotein cholesterol were adjusted. Remnant-C, remnant cholesterol.

The results from the regression analyses are described in Table 2. In the unadjusted model, Remnant-C was positively correlated to the 10-year risk of a first hard ASCVD event (β = 0.047, [95%CI: 0.007–0.087], *P* = 0.02043). After adjustment for gender, age and race, this positive association was still obvious in model 2 [(β = 0.144, 95%CI: 0.117–0.171], *P* < 0.001). After adjusted for gender, age, race, BMI, SBP, DBP, hypertension treatment, smoking, diabetes, LDL-C, the 10-year risk was increased by 0.078% for each 1 mg/dl increase in Remnant-C (β = 0.078, [95%CI: 0.061–0.094], *P* < 0.001). Compared with quartile 1 (the lowest quartiles) of Remnant-C, the 10-year risk of a first hard ASCVD event increased along with rising levels of Remnant-C as 0.202 (Q2, 95%CI, −0.475–0.879), 1.363 (Q3, 95%CI, 0.693–2.033), and 2.420 (Q4, 95%CI, 1.734–3.107) in model 3 (*P* for trend <0.001). We also performed generalized additive models and smooth curve fittings to evaluate the associations between Remnant-C and the 10-year risk (Figure 1). There was a linear relationship between Remnant-C and the 10-year risk—with increased Remnant-C, the 10-year risk increased.

**Table 2:** Association between Remnant-C and 10-year risk of a first hard ASCVD event.

	**Model 1**	**Model 2**	**Model 3**
	**β (95% CI), *P*-value**	**β (95% CI), *P*-value**	**β (95% CI), *P*-value**
Remnant-C (mg/dL)	0.047 (0.007, 0.087), *P* = 0.02043	0.144 (0.117, 0.171), *P* < 0.00001	0.078 (0.061, 0.094), *P* < 0.00001
**Remnant-C (quartiles)**			
Q2 vs. Q1	−0.503 (-2.154, 1.147), *P* = 0.55025	0.900 (-0.196, 1.996), *P* = 0.10762	0.202 (-0.475, 0.879), *P* = 0.55895
Q3 vs. Q1	1.228 (-0.363, 2.819), *P* = 0.13049	3.309 (2.239, 4.379), *P* < 0.00001	1.363 (0.693, 2.033), *P* = 0.00007
Q4 vs. Q1	2.144 (0.546, 3.742), *P* = 0.00861	5.110 (4.019, 6.201), *P* < 0.00001	2.420 (1.734, 3.107), *P* < 0.00001
*P* for trend	0.002	<0.001	<0.001

*Model 1 adjust for: none*.

*Model 2 adjust for: gender, age, race. Model 3 adjust for: gender, age, race, body mass index, systolic blood pressure, diastolic blood pressure, hypertension treatment, smoking, diabetes, low density lipoprotein cholesterol*.

*Remnant-C, remnant cholesterol; CI, confidence interval*.

### Stratified Associations Between Remnant-C and the 10-Year Risk of a First Hard ASCVD Event

**Figure 2:**
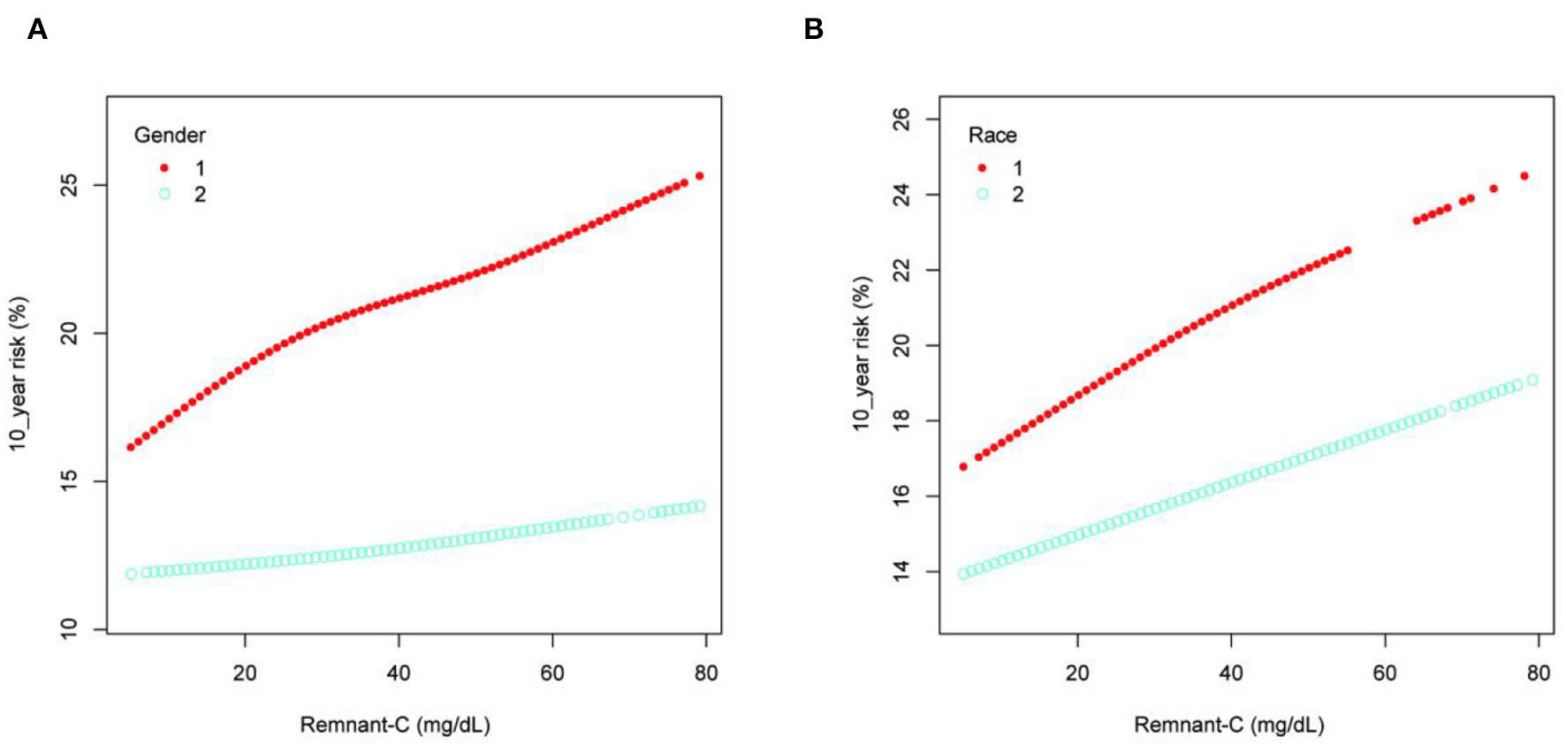
Stratified association between Remnant-C and the 10-year risk of a first hard ASCVD event. **(A)** stratified by gender, 1 = Men, 2 = Women. **(B)** stratified by race, 1 = Non-Hispanic Black, 2 = Others. Gender, age, race, body mass index, systolic blood pressure, diastolic blood pressure, hypertension treatment, smoking, diabetes, and low-density lipoprotein cholesterol were adjusted. In the subgroup analysis stratified by gender or race, the model is not adjusted for the stratification variable itself. Remnant-C, remnant cholesterol.

Subgroup analyses stratified by gender and race in Table 3, showed the positive correlation of Remnant-C with 10-year risk of a first hard ASCVD event in both men (β = 0.128, [95%CI: 0.108–0.148], P < 0.001) and women (β = 0.043, [95%CI: 0.016–0.070], *P* = 0.00179), as well as in non-Hispanic Black people (β = 0.077, [95%CI: 0.046–0.108], *P* < 0.001) and other races (β = 0.080, [95%CI: 0.061–0.094], *P* < 0.001). Smooth curve fittings and generalized additive models were used to characterize the non-linear relationship between Remnant-C and the 10-year risk of a first hard ASCVD event (Figure 2). And the result showed that Remnant-C was linearly associated with a 10-year risk in both men and women; while Remnant-C reached a saturation curve in non-Hispanic Black people. We further examined the threshold effect of Remnant-C on the 10-year risk of a first hard ASCVD event using two-piecewise linear regression models and found that the inflection point identified as 38 mg/dL (Table 4). For non-Hispanic Black people, when the Remnant-C <38 (mg/dl), the 10-year risk increased by 0.118% for each 1 mg/dl increase in Remnant-C (β = 0.118, [95%CI: 0.068–0.168], *P* < 0.001), while when the Remnant-C > 38 (mg/dl), the 10-year risk decreased with Remnant-C (β = −0.003, [95%CI:−0.086–0.080], *P* = 0.9403). The log likelihood ratio is 0.040, which means that there is a significant difference between the standard linear model and the two-piecewise linear model. The two-piecewise linear model is more suitable and the inflection point exists significantly.

**Table 3:** Effect size of Remnant-C on 10-year risk of a first hard ASCVD event in subgroup analysis stratified by gender or race.

	**Model 1**	**Model 2**	**Model 3**
	**β (95% CI), *P*-value**	**β (95% CI), *P*-value**	**β (95% CI), *P*-value**
**Subgroup analysis stratified by gender**			
Men	0.008 (-0.046, 0.062), *P* = 0.76687	0.185 (0.148, 0.222), *P* < 0.00001	0.128 (0.108, 0.148), *P* < 0.00001
Women	0.080 (0.027, 0.133), *P* = 0.00319	0.113 (0.073, 0.152), *P* < 0.00001	0.043 (0.016, 0.070), *P* = 0.00179
**Subgroup analysis stratified by race**			
Non-Hispanic Black	0.178 (0.087, 0.268), *P* = 0.00013	0.210 (0.138, 0.282), *P* < 0.00001	0.077 (0.046, 0.108), *P* < 0.00001
Other	0.049 (0.004, 0.094), *P* = 0.03344	0.131 (0.103, 0.159), *P* < 0.00001	0.080 (0.061, 0.094), *P* < 0.00001

*Model 1 adjust for: none*.

*Model 2 adjust for: gender, age, race. Model 3 adjust for: gender, age, race, body mass index, systolic blood pressure, diastolic blood pressure, hypertension treatment, smoking, diabetes, low density lipoprotein cholesterol*.

*In the subgroup analysis stratified by gender or race, the model is not adjusted for the stratification variable itself. CI, confidence interval*.

**Table 4:** Threshold effect analysis of Remnant-C on 10-year risk of a first hard ASCVD event in non-Hispanic blacks using the two-piecewise linear regression model.

**10-year risk of a first hard ASCVD event**	**Adjusted β (95% CI), P value**
**Non-Hispanic black**	
Fitting by the standard linear model	0.077 (0.046, 0.108) <0.0001
**Fitting by the two-piecewise linear model**	
Inflection point	38
remnant.c <38 (mg/dl)	0.118 (0.068, 0.168) <0.0001
remnant.c> 38 (mg/dl)	−0.003 (−0.086, 0.080) 0.9403
Log likelihood ratio	0.040

*Gender, age, body mass index, systolic blood pressure, diastolic blood pressure, hypertension treatment, smoking, diabetes and low density lipoprotein cholesterol were adjusted. ASCVD, atherosclerotic cardiovascular disease; CI, confidence interval; Remnant-C, remnant cholesterol*.

## Discussion

In this study, we used 2,048 representative samples of NHANES 1999–2018 aged from 40 to 79 to evaluate the associations between Remnant-C and the 10-year risk of a first hard ASCVD event in adults. The results revealed a positive association between Remnant-C and the 10-year risk of a first hard ASCVD event. After adjusting for gender, age, race, BMI, SBP, DBP, hypertension treatment, smoking, diabetes, LDL-C, the 10-year risk increased by 0.078% for each 1 mg/dl increase in Remnant-C (β = 0.078, [95%CI: 0.061–0.094], *P* < 0.001). However, in non-Hispanic Black people, the 10-year risk of a first hard ASCVD event increased with Remnant-C up to the inflection point (38 mg/dL). When the Remnant-C <38 (mg/dl), the 10-year risk increased by 0.118% for each 1 mg/dl increase in Remnant-C (β=0.118, [95%CI: 0.068–0.168], *P* < 0.001). When Remnant-C >38 (mg/dl), the 10-year risk decreased with Remnant-C (β = −0.003, [95%CI: −0.086–0.080], *P* = 0.9403). The log likelihood ratio is 0.040.

Atherosclerosis begins with damage of intima. During the hydrolysis of TRL, lipoproteinlipase (LPL) releases free fatty acids and monacylglycerol, which trigger local inflammation, and accelerate the formation and progression of atherosclerosis (12). The elevation of RLP in blood increases the infiltration of the arterial wall and accumulates in the arterial wall after transporting through the endodermis. Then RLP will be engulfed by macrophages and smooth muscle cells will evolve into foam cells and become part of the atherosclerotic plaque. Meanwhile, RLP increases the production of reactive oxygen species and induces endothelial cell dysfunction (13). Because Remnant-C is more numerous and bulky than LDL-C, it carries more cholesterol, and thus becomes more atherogenic (14).

In recent years, several cohort studies of primary and secondary prevention examined the association between Remnant-C levels and ASCVD. A prospective study of 25,480 patients from the Copenhagen General Population Study during a median 11 years of follow up found that VLDL cholesterol explained 50% of the myocardial infarction risk from elevated apoB-containing lipoproteins, whereas VLDL triglycerides did not explain risk (15). A 12-year follow up of 5,414 patients with ischemic heart disease (IHD) from the IHD Research Center in Copenhagen found that higher Remnant-C levels were associated with all-cause mortality (16). Bittencourt et al. found that in a longitudinal cohort study of 3,845 patients from Brazil, the increase in Remnant-C was associated with a coronary artery calcification score in a population with no previous history of cardiovascular disease. Every one standard deviation increase in Remnant-C, coronary artery calcification was associated with a 20% increase in risk (OR = 1.20, [95%CI 1.08 to 1.32], P = 0.001), suggesting Remnant-C can be used as a predictor of subclinical atherosclerosis (17). Elshazly et al. analyzed the changes in the percentage of atherosclerotic volume and major adverse cardiovascular events at 2 years at different levels of Remnant-C from 5,754 patients with coronary heart disease who underwent an intravascular ultrasound, indicating that Remnant-C is positively associated with the progression of coronary atherosclerosis volume in ASCVD patients treated with statins, independent of dyslipidemia, C-reactive protein or clinical risk factors (18). Castañer et al. analyzed the relationship between lipid levels and major adverse cardiovascular events (myocardial infarction, stroke, or cardiovascular death) in the PREDIMED cohort of high-risk cardiovascular disease groups, showing that every increase in 0.26 mmol /L of Remnant-C was associated with an increase in the risk of cardiovascular events by 21%. After multivariable adjustment, TG and Remnant-C, rather than LDL-C, were associated with cardiovascular events in individuals who were overweight or obese, independent of lifestyle and other factors. When Remnant-C>0.65 mmol/L, the volume of atherosclerotic plaque gradually increased; Remnant-C >0.78 mmol/L was considered a high-risk threshold for ASCVD (19). In our study, we found that after adjustment for confounders including gender, age, race, BMI, SBP, DBP, hypertension treatment, smoking, diabetes and LDL-C, for every 1 mg/dL increase in Remnant-C, the 10-year risk of a first hard ASCVD event in adults aged from 40 to 79 years increased by 0.078% ([95%CI: 0.061–0.094], *P* < 0.001). The advantage of our study over previous studies is that Remnant-C can also be used as a predictor for people who do not have cardiovascular disease.

There were several limitations to our study. As a secondary analysis, we cannot reach the total covariates, for example, atherosclerotic history, because some data were not collected. Likewise, after the exclusion of those without exposure factors or outcome indicators and those who did not meet the inclusion criteria, the exclusion was not based on the weight proportion, so it could not represent the whole sample of the United States. In addition, according to the method of calculating the 10-year risk of a first hard cardiovascular event, racial groups were divided into non-Hispanic Black people and others, so the particularity of other categories cannot be calculated. Finally, because the criteria for the 10-year-risk calculation had to meet many conditions, such as, participants had to be aged from 40 to 79 years, HDL 20–100 mg/dl, TC 130–320 mg/dl and SBP 90–200 mmHg, the 10-year risk could not be calculated if the conditions exceeded the numerical range.

## Conclusions

Remnant-C was positively associated with the 10-year risk of a first hard ASCVD event. This association was persistent after adjustment for confounders. However, the results of this study warrant future study.

## Data Availability Statement

Publicly available datasets were analyzed in this study. This data can be found here: https://www.cdc.gov/nchs/nhanes/index.htm.

## Author Contributions

HQ and DS: conceptualization and supervision. ZY: writing initial manuscript. KY: statistics and analyses. JS, QY, YZ, and JG: review and editing. All authors contributed to the article and approved the submitted version.

## Funding

This work was supported by Young scholar of China association for science and technology (No. 2020-QNRCI-02), Fundamental research funds for the central public welfare research institutes (No. ZZ15-YQ-006), and the project of National Natural Science Foundation of China (Grant No. 81774141).

## Conflict of Interest

The authors declare that the research was conducted in the absence of any commercial or financial relationships that could be construed as a potential conflict of interest.

## Publisher's Note

All claims expressed in this article are solely those of the authors and do not necessarily represent those of their affiliated organizations, or those of the publisher, the editors and the reviewers. Any product that may be evaluated in this article, or claim that may be made by its manufacturer, is not guaranteed or endorsed by the publisher.
